# Development of Biocomposites with Antioxidant Activity Based on Red Onion Extract and Acetate Cellulose

**DOI:** 10.3390/antiox4030533

**Published:** 2015-08-03

**Authors:** Carol López de Dicastillo, Rosa Navarro, Abel Guarda, Maria José Galotto

**Affiliations:** Center for the Development of Nanoscience and Nanotechnology (CEDENNA)-Food Packaging Laboratory (LABEN-CHILE), Department of Science and Food Technology, Faculty of Technology, University of Santiago de Chile, Santiago 9170201, Chile; E-Mails: rosa.navarro.lisboa@gmail.com (R.N); abel.guarda@usach.cl (A.G.); maria.galotto@usach.cl (M.J.G.)

**Keywords:** red onion, antioxidant capacity, active packaging, extraction, natural compounds

## Abstract

Antioxidant biocomposites have been successfully developed from cellulose acetate, eco-friendly triethyl citrate plasticizer and onion extract as a source of natural antioxidants. First, an onion extraction process was optimized to obtain the extract with highest antioxidant power. Extracts under absolute ethanol and ethanol 85% were the extracts with the highest antioxidant activity, which were the characterized through different methods, DPPH (2,2-diphenyl-1-picrylhydrazyl) and ABTS (2,2ʹ-azinobis(3-ethylbenzothiazoline-6-sulphonate)), that measure radical scavenger activity, and polyphenolic and flavonoid content. Afterwards, the extract was incorporated in cellulose acetate as polymer matrix owing to develop an active material intended to oxidative sensitive food products packaging. Different concentrations of onion extract and plasticizer were statistically studied by using response surface methodology in order to analyze the influence of both factors on the release of active compounds and therefore the antioxidant activity of these materials.

## 1. Introduction

Active food packaging appears as a promising technology with increasing applications due to its advantages over traditional methods on food preservation. Active packaging (AP) is defined as a package system that deliberately incorporates components that release or absorb substances into or from the packaged food or the environment surrounding the food to extend the shelf life or to maintain or improve the condition of the packaged food (Regulation CE No. 450/2009). Antioxidant active packaging is one of the most interesting components of AP because oxidation is one of the main problems on the preservation of oxygen sensitive food products, and recently the demand for natural antioxidant active packaging is increasing enormously due to its advantages compared with the addition of antioxidants directly to the food [1,2,3]. Nowadays, the demand for natural compounds is increasing due to consumers’ preference, and due to their lower toxicity and higher safety. Several essential oils, such as oregano, rosemary and ginger essential oils, and plant extracts, such as green tea, cocoa and grape seed extract, have been already incorporated in polymer matrices resulting on interesting results [4,5,6,7]. In addition, nowadays there is a growing interest in the substitution of traditional plastic materials by biodegradable polymers, due to serious environmental problem caused by massive accumulation of plastic waste. As a result, cellulose, one of the most abundant natural polymers, and its derivatives, are attracting interest because of their easy processability. Cellulose acetate (CA) is of particular interest due to its excellent optical clarity and high toughness, and because this polymer can be processed by solvent-casting and melting techniques [8,9].

Therefore, the aim of this study was the development of an antioxidant active packaging based on acetate cellulose, as biodegradable polymer matrix, and red onion extract, as a source of antioxidant compounds. Onions (*Allium cepa*) are botanically included in the *Lilliaceae* and, after tomatoes, are the second most horticultural vegetable [10]. Furthermore, onions are one of the main sources of dietary polyphenols in many countries [11]. Quercetin is the most common flavonol aglycone, although it is usually present as glycosides, quercetin-3,4-diglucoside and quercetin-4-glucoside being the most abundant, which make up to 80%–85% of the total flavonoid content. Red onions have the highest levels of flavonoids, followed by the yellow cultivars. They also display anthocyanins, which not only impart red color but also participate in their strong antioxidant activity [12,13]. *In vitro* studies have shown that flavonoids compounds may have positive effects against inhibition oxidation of biomolecules by its antioxidative potential, anti-inflammatory, anti-allergic, and antifungal effect [14,15,16].

There are two main mechanisms of action for antioxidant packages: (i) the scavenging of undesirable compounds that are involved in any step of the oxidation process, such as oxygen, radical oxidative species or metal ion from the headspace or from the food; and (ii) the technologies based on the release of antioxidants to the food. The release of the active agent is dependent on several factors, such as polymer type and food, and concentrations of the bioactive agents, among others. In this case, the design of active packages must involve the positive interaction between package and packaged food or headspace atmosphere [17,18]. Therefore, an important factor to be considered in the packaging design is the plasticizer. This is a component generally used with the aim of reducing the film’s stiffness by weakening intermolecular forces and improving the molecular chain mobility. All these properties affect the strength and integrity of the casting films, further affecting the performance of the release of the active compound. Otherwise, recent studies have incorporated plasticizers with the aim of controlling active compounds release [19,20,21]. Triethyl citrate, TEC, was selected as plasticizer because it is eco-friendly, maintaining the biodegradability of the biocomposite [22]. Different concentrations of plasticizer were formulated in order to study the effect on the release rate of antioxidant components from active material on food simulants.

Several workers have used surface response for designing food formulations and processing and optimization of different method [23,24,25]. Response surface methodology (RSM) has been reported to be an effective tool for optimizing a process. The main idea of RSM is to use a sequence of designed experiments to obtain an optimal response. RSM is used commonly for experimental design, model building, to obtain an optimal conditions and consideration of different factors [26].

Considering that the several parameters employed during film production influence film properties, the present study aimed to determine the optimal formulation by using response surface methodology in order to obtain films with activity antioxidant and evaluate kinetic release behavior of these materials.

## 2. Materials and Methods

### 2.1. Chemicals and Reagents

Cellulose acetate (39.8% weigth acetyl content. Mn *ca.* 30.000) was supplied by Aldrich. Reagent-grade absolute ethanol, quercetin dihydrate, 2,2-diphenyl-1-picrylhydrazyl (DPPH) radical, 2,2ʹ-azinobis(3-ethylbenzothiazoline-6-sulphonate) (ABTS), Folin Ciocalteau phenol reagent, anhydrous sodium carbonate, gallic acid, 6-hydroxy-2,5,7,8-tetramethylchroman-2-carboxylic acid (Trolox), and triethyl citrate (>99%) (TEC) were purchased from Sigma Aldrich Quimica S.A. (Santiago, Chile). Red onions were bought at a local supermarket in Santiago, Chile. Owing to avoiding differences in batches, the starting materials were all bought at the same time.

### 2.2. Red Onion Extraction Studies

#### 2.2.1. Extraction of Active Compounds from Red Onion

First, a portion of red onion was extracted using different solvent systems: acetone, water, ethanol 75% and 85%, and absolute ethanol, aiming to obtain the extract with highest antioxidant activity. Approximately, 15 g of chopped fresh red onion was stirred with 40 mL of solvent at 40 °C and 150 rpm for 90 min. Later, the extracts were centrifuged and used for analysis through DPPH method. The two extractions with highest DPPH scavenging activity were selected for further study through other antioxidant ability assays and polyphenolic content.

#### 2.2.2. Determination of Polyphenolic Compounds

The total phenolic content was estimated colorimetrically by means of the Folin-Ciocalteu method with slight modifications [11,27]. A volume of 100 μL of the extract was mixed with 3100 μL of distilled water and 200 μL of Folin–Ciocalteu reagent. The mixture was shaken and stored in the dark for 5 min, and, subsequently, 600 μL of anhydrous sodium carbonate (20% v/v) was added and stirred until homogeneous color was observed. After 2 h of reaction, the coloration of the samples was measured at an absorbance of 765 nm. Gallic acid was used as the standard to express the results as mg gallic acid/100 g of fresh sample. The experiments were performed in triplicate.

#### 2.2.3. Evaluation of Antioxidant Capacity of Extracts

Because the antioxidant activity of natural extracts are attributed to several mechanisms, the antioxidant power of onion extract was measured by three different antioxidant assays: DPPH, ABTS and FRAP (ferric reducing antioxidant power) methods, which are often used because they are simple, inexpensive and robust techniques.

FRAP assay is a total electron transfer based method and measures reduction of ferric 2,4,6-tripyridyl-*s*-triazine (TPTZ) to a colored product, so, in combination with other methods, it can be very useful in distinguishing dominant mechanisms with different antioxidants [28]. DPPH and ABTS assays measure the antioxidant effectiveness by monitoring the inhibition of oxidation of a suitable substrate, which may be neutralized either by direct reduction via electron transfers or by radical quenching via H atom transfer.

DPPH· radical-scavenging activity of onion extracts was evaluated according to the method described by Okada and Okada with some modifications [29]. About 5 mL of sample was incubated with DPPH· solution for 30 min in the dark at room temperature, and the absorbance was determined at 517 nm. ABTS^•+^ radical cationic solution were produced by reacting 7 mM ABTS in water with 2.45 mM potassium persulfate (K_2_S_2_O_8_) and then stored in darkness at room temperature for 16 h. ABTS^•+^ radical solution was diluted to give an absorbance value of 1 at 734 nm. All experiments were performed in triplicate. In both assays, the percentage inhibition values were calculated using this Equation (1):

I (%) = (A_control_ − A_sample_)/A_control_ × 100 (1)

where A_sample_ represents the sample absorbance and A_control_ the control sample absorbance (only solvent, no antioxidant included). To standardize the results, scavenging activities of the DPPH· and ABTS^•+^ radicals were expressed as Trolox equivalents using a calibrated curve of Trolox concentration *versus* I (%).

FRAP assay was measured using 150 μL of onion extract in accordance with Lu *et al.* (2011) studies [30]. The FRAP reagent was prepared from 2.5 mL of a TPTZ solution (10 mmol/L) in hydrochloric acid (40 mmol/L) and 2.5 mL of a FeCl_3_ solution (20 mmol/L) mixed with 25 mL of an acetate buffer (0.3 mol/L, pH 3.6). The reaction mixture was stored at room temperature for 30 min and the absorbance was measured at 593 nm. Results were expressed as mg Trolox/100 g sample.

#### 2.2.4. Determination of Total Flavonoid Content

Total flavonoid content was measured by the aluminum chloride colorimetric assay, following the method described by Albishi *et al.* [31]. One milliliter of extract or standard solution of quercetin was added to 10 mL volumetric flask containing 4 mL distilled water. Then, 0.3 mL of 5% NaNO_2_ solution was added, and after 5 min, 0.3 mL 10% AlCl_3_ was added. After 6 min, 2 mL 1 M NaOH solution was added and the total volume was made up to 10 mL with distilled water. The solution was well mixed and the absorbance was measured at a 510 nm. Total flavonoid content was expressed as mg quercetin/100 g fresh sample.

### 2.3. Obtaining Onion Extract (OE)

In order to obtain an extract with higher concentration to be incorporated for film development, onion was extracted with ethanol (3 g:20 mL) at 40 °C during 3 h. Solution was filtered and concentrated to a final concentration of 600 mg onion/ml extract.

### 2.4. Films Preparation

Films were obtained by a solution–extension–evaporation process (“casting”), containing cellulose acetate, triethyl citrate, TEC, as plasticizer and onion extract. Then, 2.5 g of polymer was dissolved in 50 mL acetone at 50 °C under stirring. As Table 1 shows, different concentrations of the onion extract and plasticizer were incorporated in order to achieve the optimum formulation for the active material. Casting was done over a petri dish of 18 cm diameter and film drying was accomplished by using a stove at 40 °C for approximately 2 h. The thickness of every sample was individually measured using a digital micrometer with an average value of (170 ± 5) μm.

**Table 1:** Independent variables and levels used for surface response composite central design.

**Independent Variables**	**Levels**				
	−14.142	−1	0	1	14.142
OE concentration (%) (X1)	3.964	5	7.5	10	11.035
Plasticizer concentration (%) (X2)	13.786	20	35	50	56.213

Finally, films were stored at room temperature in vacuum packages until further analysis. The total antioxidant activity of the films was determined by extractions in ethanol using ultrasonic at room temperature. Resulting film extracts were analyzed using DPPH method. Blank samples (films without OE incorporated) were also analyzed.

### 2.5. Antioxidant Release Test

Release studies of the active compounds from the films were carried out by determining the migration from the polymer into 10% ethanol solution, as aqueous food simulant, at 40 °C. Double-sided total immersion migration tests were performed as follows: a 3 cm^2^ piece of each plastic sample and 5 mL of the simulant (area-to-volume ratio 6 dm^2^/L) were placed in tubes. Periodically, vials were opened and the concentration of the antioxidants in the aqueous simulants was analyzed by indirect measurement through DPPH scavenging activity assay (explained at Section 2.2.3). To standardize the results, antioxidant activities of simulants were expressed as Trolox equivalents using a calibrated curve of Trolox concentration *versus* I (%).

### 2.6. Statistical Analysis

Fisher’s least significant difference (LSD) procedure was used to evaluate statistically significant differences at the 95.0% confidence level, between the samples extracted with different solvents.

Response surface methodology (RSM) was used in this experiment to study the effects of plasticizer and onion extract concentration on the antioxidant activity of film material (*i.e.*, antiradical activity and total polyphenol). A Central Composite Rotatable Design (CCRD) including 18 experiments formed by 4 central points and 4 (*k* = 1.41421) axial points to 2^2^ full factorial design was used. Two levels for each variable were selected: plasticizer: 20%–50% respect to polymer weight, and onion extract: 5%–10% respect to polymer weight. Total phenolic content (TPC) and antioxidant capacity (expressed as Trolox equivalents) were chosen as the response variables. The behavior of the response surface was fitted to a second order polynomial model, according to Equation (2):

(2) $$Y=\;\beta_{0}+\beta_{1}x_{1}+\beta_{2}x_{2}+\beta_{11}x_{1}^{2}+\beta_{22}x_{2\;}^{2}+\beta_{12}x_{1}x_{2}$$

where *Y* is the calculated response by the model; β_0_ is a constant intercept; β*_i_* is lineal; and β*_ii_* and β*_ij_* are squared and interaction coefficient, respectively. Optimization procedures were performed using Statgraphics Centurión XV (StatPoint Inc., Warrenton, VA, USA). The quality of the fit of the polynomial model equation was evaluated by adjusted coefficients of determination (*R*^2^_adj_), standard error of estimation and lack of fit, according to Myers and Montgomery (2002) [32].

The range and coded level of the red onion extract concentration and plasticizer concentration as process variables studied are listed in Table 1. The choices of range were selected based on preliminary studies in our laboratory and it was necessary to apply a process to concentrate the extract. The complete design matrix of composite central design and the experimental results are given in Table 2.

## 3. Results and Discussion

### 3.1. Characterization of Onion Extract

First, multiple extractions under different solvents were carried out in order to select the best solvent to extract most antioxidant compounds. In general, the antioxidant activity varied in the red onion extracts and ranged from 6.5 to 9.7 mg Trolox/100 g of sample material, according the solvent used for the extraction. The data for DPPH radical-scavenging activity values are shown in Table 3. On the other hand, extractions done with acetone and ethanol 75% exhibited significantly higher DPPH radical-scavenging activity than water (*p* = 0.0213).

Once 85% ethanol and absolute ethanol were selected as the two best solvents, these two extracts were analyzed through other methods. It is recommended to use various methods for evaluating the antioxidant capacity of complex heterogeneous systems like foods, as no general standardized protocol is currently available due to the dependence of the antioxidant activity on the chemical structure, mechanisms and antioxidant’s chemical environment [28].

**Table 2:** Experimental data with coded and actual values of variables and levels.

Experiment	Factors		Actual Responses		Predicted Responses	
	OE concentration	Plasticizer	DPPH	Total Phenols	DPPH	Total Phenols
	(%)	(%)	(mg Trolox/g of Sample)	(mg GAE/g of Sample)	(mg Trolox/g of Sample)	(mg GAE/g of Sample)
1	5	20	0.355	1.303	0.331	1.28
2	10	20	0.854	2.034	0.858	1.94
3	5	50	0.357	0.977	0.416	0.976
4	10	50	0.212	1.525	0.298	1.453
5	3.96	35	0.456	0.945	0.445	0.942
6	11.04	35	0.787	1.648	0.735	1.746
7	7.5	13.79	0.502	1.696	0.529	1.759
8	7.5	56.21	0.283	1.168	0.194	1.2
9	7.5	35	0.489	1.341	0.509	1.427
10	7.5	35	0.56	1.525	0.509	1.427
11	7.5	35	0.479	1.339	0.509	1.427
12	7.5	35	0.508	1.502	0.509	1.427

**Table 3:** Comparison of antioxidant activity contents in onions above different extraction solvent.

Solvent	mg Trolox/100 g Sample
Acetone	9.7 ± 0.4 ^a^
Water	6.5 ± 0.3 ^b^
Ethanol 75%	9.5 ± 0.9 ^a^
Ethanol 85%	13.1 ± 0.5 ^c^
Ethanol	17.0 ± 0.7 ^d^

Letters ^a–d^ indicate significant differences in antioxidant activity among different extracts.

Extracts were evaluated through the following assays: DPPH and ABTS to measure radical-scavenging activity, and FRAP to measure ferric reducing power of extracts. Polyphenolic and flavonoid content were also determined through Folin Cicolteau and aluminum chloride colorimetric assay, respectively.

As Table 4 shows, results indicated that the sample containing absolute ethanol resulted statistically different and showed the highest values in practically all properties measured, except in flavonoids content. Specifically, the DPPH radical-scavenging activity and total polyphenol content results presented the major variation.

**Table 4:** Antioxidant activities, polyphenolic and flavonoid content of red onion extracts measured through different methods.

Method	Ethanol 85%	Ethanol 100%
Total Phenols (mg GAE/100 g of sample)	232.2 ± 2.5 ^a^	287.6 ± 5.0 ^b^
ABTS (mg Trolox/100 g of sample)	109.1 ± 2.4 ^a^	118.3 ± 0.2 ^b^
FRAP (mg Trolox/100 g of sample)	57.6 ± 0.9 ^a^	59.1 ± 1.0 ^a^
Flavonoid (mg quercetin/100 g of sample)	187.8 ± 2.1 ^a^	178.7 ± 5.3 ^a^

Lower case letters ^a,b^ indicate significant differences in method results among the extracts.

These results were in accordance with those of Kahkonen *et al.* [33] who detected total phenolics of 2500–3000 GAE mg/kg in onion and Nuutila *et al.* [11], who determined that the amounts of phenolics varied between 1928 and 2222 GAE mg/kg in red onion. Moreover, they reported that the total quercetin content of dry skin of red onion was also approximately twice that of the dry skin of yellow onion. The selected flavonoids are well known by their antioxidant activity, which is due to their ability to chelate metal ions and to reduce oxidative radicals [11,33].

### 3.2. Experimental Design for Film Formulation

Response surface methodology was carried out owing to estimate the optimal formulation of the films with the incorporation of red onion extract obtained. A central composite design was employed to evaluate the DPPH radical-scavenging activity and Total Phenol after the formulation of different films. The statistical combination of the independent variables in actual values along with the predicted and experimental response was presented in Table 2.

The independent and dependent variables were fitted by the second-order polynomial equation to the experimental data. The model expressed by Equations (3) and (4) represents DPPH (*Y*) and Total phenols as a function of red onion concentration extract (*X*1) and plasticizer concentration (*X*2). In both cases, there was no significant (*p* > 0.05) lack-of-fit, which indicated that the response models were accurately fitted for predicting the responses as function of independent variables studied (Table 5).

DPPH = −0.687 + 0.094 × [*X*1] + 0.047 × [*X*2] + 0.006 × [*X*1]^2^ − 0.004 × [*X*1] × [*X*2] − 0. 0003 × [*X*2]^2^ (3)

Total Phenols = 0.487 + 0.255 × [*X*1] − 0.012 × [*X*2] − 0.006 × [*X*1]^2^ − 0.001 × [*X*1] × [*X*2] + 0.0001 × [*X*2]^2^ (4)

The statistical significance was analyzed by Fisher test and the analysis of variance (ANOVA) for each response variable, which is given in Table 5. Values of probability *p* > F less than 0.05 indicate that model terms are significant. DPPH radical-scavenging activity and total phenol were notably (*p* > 0.05) affected by the concentration of plasticizer and red onion extract (Table 5). It is may be due to the fact that these plasticizers perform different outcomes based on their intrinsic properties and the interaction with polymers [34]. In general, the interaction effect of plasticizer and onion extract had a negative effect on the responses variables, thus indicating the positive close correlation between DPPH radical-scavenging activity and Total phenols. However, this interaction resulted significant just for DPPH radical-scavenging activity. Also, the quadratics effects resulted significant different respect DPPH radical-scavenging activity.

**Table 5:** Regression coefficients of the second-order polynomial response models.

Source	DPPH		Total Phenols	
	F-Ratio	*p*-Value	F-Ratio	*p*-Value
[*X*1]	64.02	0.004	64.88	0.000
[*X*2]	85.71	0.002	31.38	0.001
*X*1 *X*1	7.97	0.066	1.09	0.336
*X*1 *X*2	78.92	0.003	0.84	0.394
*X*2 *X*2	26.51	0.014	0.45	0.528
Lack-of-fit	5.81	0.091	0.96	0.514
*R*^2^_aj_	87.12		89.52	
SEE	0.06		0.10	

The coefficient of determination (*R*^2^_aj_) indicated that the accuracy of the model obtained from the ANOVA was quite good; whose values were 0.90 and 0.87 for total phenol and DPPH activity, respectively. These results suggest that the models fitted for the antioxidant activity and total phenols are suitable (significant and predictive) and lead to significant regression, low residual values, no lack of fit, and satisfactory coefficients of determination (Table 5).

**Figure 1:**
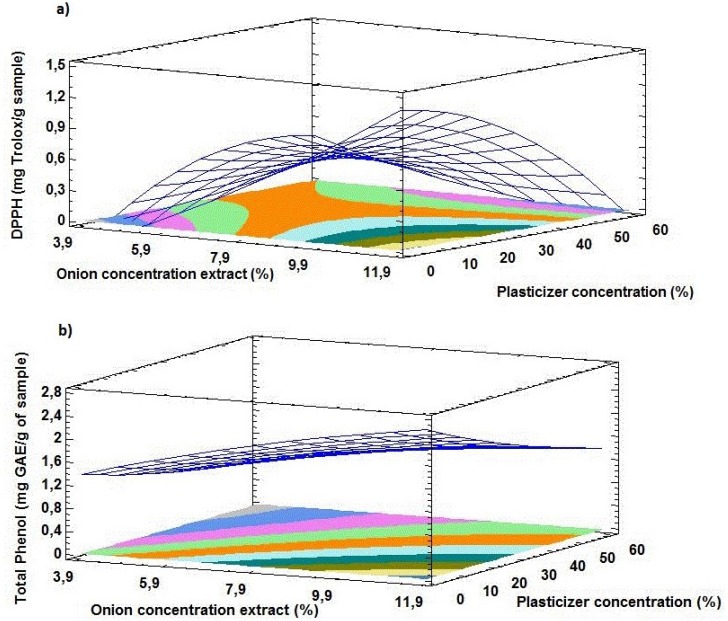
The surface plots of (**a**) total phenol and (**b**) DPPH radical-scavenging activity of forming film process by the plasticizer and onion extract.

The polynomial response models were expressed as three-dimensional (3D) surface plots to better visualize the relationship between the plasticizer and onion extract processing conditions as independent variables and antioxidant activity and total phenol as response variables (Figure 1). Graphical optimization was carried out to determine the optimum condition of film forming process leading to favorable release active agents from cellulose acetate based films in terms of DPPH antioxidant activity and total phenol. Figure 1a shows that the combination of factor levels, which minimized total phenol was produced by performing the low onion extract (3.9%) and high plasticizer concentration (56.2%). On the other hand, Figure 1b shows the DPPH activity performed at high onion extract (11%) and high plasticizer concentration (56.2%) provided the film with lowest release antioxidant agent in comparison with other film formulated.

Finally, regarding to the results of blank samples, there were no antioxidant activity observed.

### 3.3. Antioxidant Compounds Release

Release of active agents from developed materials to an aqueous food simulant was kinetically monitored though migration assays. Antioxidant active agents released from PLA based films were indirectly measured as antioxidant activity of food simulant through DPPH method because it very well known that antioxidant activity is completely proportional to antioxidant release kinetics [6,7,35,36]. The main mechanism of action of the materials developed is through the release of the antioxidants compounds to the food product; therefore, this assay, with a food simulant, is important for understanding the future behavior of the developed materials.

**Figure 2:**
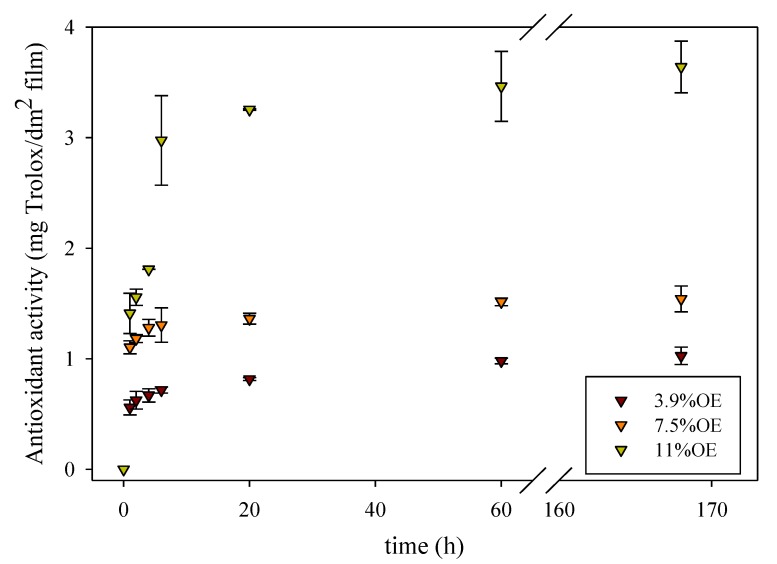
Kinetic of antioxidant activity of food simulants exposed to cellulose acetate-based films with 35% TEC (w_TEC_/w_polymer_) and three onion extract concentrations (%OE w_onion_/w_polymer_).

**Figure 3:**
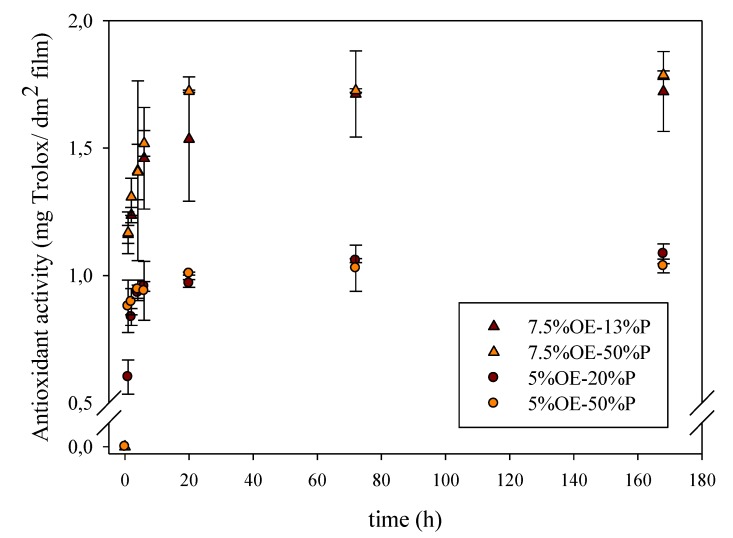
Kinetic of antioxidant activity of food simulants exposed to cellulose acetate-based films with different onion extract concentration (%OE w_onion_/w_polymer_) and plasticizer (%P).

The extent of release at equilibrium depends on the compatibility of the migrant with food simulant and the polymeric film. The higher the solubility in the simulant, the higher the release expected. Figure 2 and Figure 3 show, as an example, the influence of OE and plasticizer concentration on the antioxidant release. It was observed that the radical scavenging activity, expressed as Trolox equivalents, of the materials developed was in accordance with the antioxidant release values. All samples had a similar behavior, an “exponential growth to a maximum” type of profile, and the extent of the release was proportional to the nominal concentration of onion extract incorporated. Similar results were obtained in previous works about the release of natural extracts and compounds from hydrophilic materials [7,20,37].

Although some studies have shown release of active compounds increased with plasticizer concentration, in this work, as Figure 3 shows, the scavenging activity measured was not fully dependent on plasticizer content. At small concentrations, there were no differences between release rate and extent. Nevertheless, when concentration of OE was higher than 5% content, some differences appeared. Materials with higher concentration of plasticizer had a higher release rate, even though the extent was very similar. Polymer chains mobility increased, and antioxidants were faster released to food simulants.

Controlling OE and plasticizer concentrations, different release rates and extents can be achieved, which is incredibly interesting for future food conservation based in active food packaging.

## 4. Conclusions

It was possible to obtain biodegradable materials with antioxidant capacity for future oxygen sensitive food products conservation. A natural extract obtained from onions was successfully obtained and incorporated in cellulose acetate and the effect of onion extract and plasticizer concentration was studied through response surface methodology. The independent variables studied were plasticizer and onion extract concentrations. The results indicated that onion concentration had a significant effect on the DPPH radical-scavenging activity and total phenol (*p* > 0.05), thereby influencing the release of the antioxidant agent from the film formulated. Nevertheless, plasticizer had no significant effect on the release rate, but films with higher plasticizer concentration showed faster release due to higher mobility of the polymer chains.
